# Association Between Maternal Periodontitis and Adverse Pregnancy Outcomes: A Cross-Sectional Study at a Maternity Hospital in Kathmandu, Nepal

**DOI:** 10.7759/cureus.76544

**Published:** 2024-12-28

**Authors:** Shakti Sharma, Manita Bartaula, Subash Risal, Nishchal Devkota

**Affiliations:** 1 Department of Dentistry, Sydney Dental Clinic, Lalitpur, NPL; 2 Department of Public Health, National Open College, Lalitpur, NPL; 3 Department of Gender Studies, Tribhuvan University, Kathmandu, NPL

**Keywords:** adverse pregnancy outcome, cross sectional, maternal, periodontitis, tertiary hospital

## Abstract

Background: Adverse pregnancy outcomes, including preterm birth and low birth weight, are major global health challenges, leading to millions of newborn deaths each year. Since 1996, periodontitis and related gum diseases have been proposed as potential contributing factors, but research findings remain mixed. Further research is needed to clarify this link.

Objective: This study aims to assess the status of periodontitis and its association with adverse pregnancy outcomes.

Methodology: A cross-sectional study was conducted among 145 third-trimester mothers attending antenatal care (ANC) checkups at Maternity Hospital in Kathmandu. Data were gathered from medical records, interviews, and oral health screenings using the Community Periodontal Index (CPI) for periodontitis. Birth outcomes were assessed after delivery through medical records, which are considered highly reliable, with established protocols for data entry, consistent documentation, and regular quality control measures ensuring accuracy and consistency across healthcare providers. Ethical approval was obtained from the Nepal Health Research Council (NHRC) (approval no. 423) on September 19, 2023.

Results: Periodontitis was observed in 53 (36.6%) participants, with a higher prevalence in individuals aged 30 or more (10, 43.5%) and those who were illiterate (4, 50%). Health conditions such as hypertension, diabetes, and urinary tract infections were associated with higher periodontitis rates. However, no significant associations were found between age, education, obstetric history, or health conditions and adverse pregnancy outcomes. Periodontitis showed a significant association with adverse outcomes, with 56.6% of those with periodontitis experiencing adverse outcomes, compared to 32.6% without periodontitis (*P* = 0.005). The odds ratio of 2.69 indicates individuals with periodontitis are 2.69 times more likely to experience adverse outcomes.

Conclusions: Maternal periodontitis is significantly associated with adverse pregnancy outcomes, with individuals having periodontitis being 2.69 times more likely to experience such outcomes. These findings highlight the importance of incorporating periodontal care into prenatal healthcare.

## Introduction

Adverse pregnancy outcomes, such as preterm birth and low birth weight, are major global health concerns, with 2.3 million newborns dying within their first month each year [1]. Periodontitis has been suggested as a potential risk factor [2], initially linked to low birth weight by Offenbacher in 1996 [3]. Systematic reviews and meta-analyses have shown mixed results [4,5], with some studies failing to find a strong association [6,7]. Research from Nepal has also found correlations between maternal periodontitis and poor birth outcomes [8,9]; however, the findings are limited by insufficient analysis to determine the strength of these associations and a narrow focus on specific outcomes, such as preterm birth, without considering a wider range of adverse pregnancy outcomes. Further, a 2022 study highlighted inconsistencies in this association [10], necessitating further investigation to clarify this relationship. Our research explores a unique approach by examining multiple adverse pregnancy outcomes, expanding the scope of the inquiry to provide a more comprehensive understanding of how maternal periodontitis may influence pregnancy. This study is essential for addressing the knowledge gap regarding maternal periodontal health's impact on pregnancy outcomes in the Nepalese context, where further evidence is needed to confirm or refute these associations. The findings could have significant public health implications by informing preventive measures, such as oral health education and screenings for pregnant women, to potentially reduce the risk of adverse pregnancy outcomes. Identifying a clear link could lead to the development of targeted interventions to improve maternal and fetal health, particularly in resource-limited settings like Nepal, where oral healthcare often receives less attention. 

## Materials and methods

A cross-sectional analytical study, registration no. 3030, was used to investigate the association between maternal periodontitis and adverse pregnancy outcomes. The study was conducted at the Antenatal Care (ANC) ward of the Maternity Hospital in Kathmandu, Nepal. The study population chosen was a third-trimester pregnant woman who attended delivery at the ANC ward. Because the result/outcome of pregnancy would be known sooner, the study focused on pregnant women in their third trimester attending for delivery.

The formula used to determine the minimum sample size for a cross-sectional study is given as follows:

*n* = *z*^2^*p*(1 - *p*)/*d*^2^

where *n* is the sample size, *z* is the standard normal variate set at 95%, *p* is the prevalence of periodontitis taken from a prior study (24%) [11], *q* = 1 - *p*, and *d* is the allowable error (considering 0.07). The sample size computed was 145.

This study focused on pregnant women admitted to the ANC ward for delivery. Data collection was conducted over three months, from August to October 2023. The inclusion criteria comprised pregnant women in their third trimester, those admitted to the hospital for delivery within the study period, and those who provided informed written consent. The exclusion criteria included women admitted to intensive care or emergency units, those unwilling to participate, and women with circumstances such as alcohol or tobacco use, a history of low-birth-weight delivery or abortion, anemia, uterine tumors, syphilis, seizure disorders, or use of depression medication. Women with fewer than two teeth present or only third molars were also excluded, as were those with periodontal attachment loss attributed to non-periodontal causes such as traumatic gingival recession, cervical dental caries, distal loss of second molars, endodontic lesions draining through marginal periodontium, or vertical root fractures.

The data collection process began with a review of patient records in the ANC ward to identify eligible participants. Those who met the inclusion criteria and provided consent were administered a questionnaire covering age and educational status (Appendix). Additional information, including pregnancy and medical history, was gathered from medical records or ANC cards. Oral health status was assessed using modified WHO criteria and the Community Periodontal Index (CPI) [12]. The examination was performed by a first author as a trained dentist in a well-lit room, with participants lying on their beds for comfort [13]. A mouth mirror and CPITN probe with a 5 g weight and 0.25 N force were used to examine six index teeth, with the highest score recorded for each patient. Periodontal disease was diagnosed based on clinical attachment loss (>3 mm) and pocket depth (>4 mm) in more than two non-adjacent teeth. Those aged 20 and above had the permanent index teeth (11, 16, 17, 26, 27, 31, 36, 37, 46, 47) examined, while those below 20 had the index teeth (11, 16, 26, 31, 36, 46) examined. Depending on the most affected teeth, the highest score was assigned to each sextant (mesio-buccal/facial, mid-buccal/facial, disto-buccal/facial, peri-lingual/palatine, mid-lingual/palatine, disto-lingual/palatine) [14-16]. Following delivery, pregnancy outcomes were recorded from medical records, focusing on adverse outcomes such as low birth weight (<2,500 g), preterm birth (<37 weeks gestation), small for gestational age (birth weight below the 10th percentile for gestational age), congenital anomalies (structural or functional malformations identified before or at birth), and fetal death (occurring after 20 weeks of gestation) [17-19]. Ethical clearance for the study was obtained from the Nepal Health Research Council (NHRC), with reference number 423. Privacy and confidentiality were maintained throughout the study, and participants were assured that the information would only be used for research purposes. During the examination, a curtain was used to ensure privacy, and no visitors were allowed inside the compartment. Data were entered into Microsoft Excel and analyzed using SPSS version 16 (IBM Corp., Armonk, NY), with Chi-square tests employed to determine associations between outcome and independent variables at a 95% confidence interval (CI).

## Results

The mean age of the participants was 24.86 ± 4.63 years, with a minimum age of 18 years and a maximum of 39 years, and it has been categorized for analysis. Periodontitis was observed in 53 (36.6%) participants. Periodontitis was more prevalent with increasing age, affecting 10 (43.5%) individuals aged 30 or more compared to those under 19 (3, 21.4%). Illiterate individuals showed a higher prevalence (4, 50%) compared to literate ones (49, 35.8%). Among obstetric characteristics, individuals with more than two deliveries and cesarean deliveries had higher periodontitis rates. Health conditions like hypertension (9, 56.3%), diabetes (4, 50%), and urinary tract infections (39, 39.4%) showed a higher prevalence, while thyroid disorders showed a lower prevalence (5, 27.8%) (Table 1).

**Table 1:** Distribution of background and obstetric characteristics by the presence of periodontitis.

Background characteristics	Presence of periodontitis	
Age-wise distribution	No, 92 (63.4%)	Yes, 53 (36.6%)
Less than 19 years	11 (78.60%)	3 (21.40%)
20-29 years	68 (63.00%)	40 (37.00%)
30 or more	13 (56.50%)	10 (43.50%)
Education-wise distribution		
Illiterate	4 (50.00%)	4 (50.00%)
Literate	88 (64.20%)	49 (35.80%)
Obstetric-related characteristics		
Number of previous delivery	72 (65.50%)	38 (34.50%)
One	13 (56.50%)	10 (43.50)
Two	7 (70.00%)	3 (30.0%)
More	0 (0.0%)	2 (100.0%)
Mode of delivery		
Normal delivery	59 (67.80%)	28 (32.20%)
Cesarean delivery	33 (56.9%)	25 (43.1%)
Status of urinary tract infection		
Absent	60 (60.60%)	39 (39.40%)
Present	32 (69.60%)	14 (30.40%)
Status of hypertension		
Absent	85 (65.90%)	44 (34.10%)
Present	7 (43.80%)	9 (56.3%)
Status of diabetes		
Absent	88 (64.20%)	49 (35.80%)
Present	4 (50.00%)	4 (50.00%)
Status of thyroid disorder		
Absent	79 (62.20%)	48 (37.80%)
Present	13 (72.20%)	5 (27.80%)

Age groups

Participants were categorized into three age groups: <19 years (7, 50.0%), 20-29 years (42, 38.9%), and ≥30 years (11, 47.8%). There were no statistically significant differences in adverse outcomes among these age groups (*P* = 0.577).

Education level

The participants were also grouped by education level: illiterate (4, 50.0%) and literate (56, 40.9%). Education level did not significantly influence adverse outcomes (*P* = 0.718).

Previous delivery history

The previous delivery history did not demonstrate a clear pattern. Among the participants, 43 (39.1%) had no previous deliveries, 13 (56.5%) had one, 3 (30.0%) had two, and 1 (50.0%) had more than two deliveries. There were no statistically significant differences in adverse outcomes based on previous delivery history (p = 0.393).

Mode of delivery

Regarding the mode of delivery, 36 participants (41.4%) had a vaginal delivery, while 24 (41.4%) had a cesarean section. There was no significant difference in adverse outcomes between the two groups (p = 1.000).

Adverse outcomes were not significantly affected by the presence of hypertension, with 8 participants (50.0%) experiencing adverse outcomes compared to 52 participants (40.3%) without hypertension (*P* = 0.457). The prevalence of adverse outcomes was higher in those with diabetes mellitus (DM), with 6 participants (75.0%) affected compared to 54 participants (39.4%) without DM (*P* = 0.066). There were trends observed in urinary tract infections, with 14 participants (30.4%) affected compared to 46 participants (46.5%) without UTIs (*P* = 0.068), and in thyroid diseases, with 6 participants (33.3%) affected compared to 54 participants (42.5%) without thyroid disease (*P* = 0.459). However, none of these differences reached statistical significance (Table 2).

**Table 2:** Association of background and obstetric characteristics with adverse pregnancy outcomes. ^†^Chi-square test. ^‡^Likelihood ratio. ^#^Fisher's exact test.

Age	Adverse outcome, 60 (41.4%)	No adverse outcome, 85 (58.6%)	*P*-value
Age-wise distribution			
<19 years	7 (50%)	7 (50%)	0.577^†^
20 to 29 years	42 (38.9%)	66 (61.6%)	
30 or more	11 (47.8%	12 (52.2%)	
Education-wise distribution			0.718^#^
Illiterate	4 (50%)	4 (50%)	
Literate	56 (40.9%	81 (59.1%)	
Number of previous deliveries			0.393^‡^
No previous deliveries	43 (39.1%)	67 (60.9%)	
One	13 (56.5%)	10 (43.5%)	
Two	3 (30%)	7 (70%)	
More	1 (50%)	1 (50%)	
Mode of delivery			
Vaginal delivery	36 (41.4%)	51 (58.6%)	1.000^†^
Cesarean delivery	24 (41.4%)	34 (68.6%)	
Status of hypertension			
Absent	52 (40.3%)	77 (59.7%)	0.457^†^
Present	8 (50%)	8 (50%)	
Status of diabetes			
Absent	54 (39.4%)	83 (60.60%)	0.066^#^
Present	6 (75%)	2 (25%)	
Status of thyroid disorder			
Absent	54 (42.5%)	73 (57.5%)	0.459^†^
Present	6 (33.3%)	12 (66.7%)	
Status of urinary tract infection			
Absent	46 (46.5%)	53 (53.5%)	0.068^†^
Present	14 (30.4%)	32 (69.6%)	
Status of periodontitis			
Absent	30 (32.6%)	62 (67.4%)	0.005^†^
Present	30 (56.6%)	23 (43.4%)	

Since no associations were observed between other explanatory variables and adverse outcomes, bivariate analysis was conducted solely between periodontitis and adverse outcomes, reinforcing the importance of periodontal health as a key factor influencing adverse outcomes. The relationship between periodontitis and adverse outcomes shows that among individuals with periodontitis, 30 cases (56.6%) experienced adverse outcomes, compared to 23 cases (43.3%) who did not. In contrast, among those without periodontitis, 30 cases (32.6%) had adverse outcomes, while 62 cases (67.4%) did not. With a *P*-value of less than 0.005, the statistical analysis showed a significant relationship. The odds ratio (OR) of 2.69 suggested that individuals with periodontitis are 2.69 times more likely to experience adverse outcomes compared to those without periodontitis (Table 3).

**Table 3:** Association between periodontitis and adverse pregnancy outcome. ^†^Chi-square test. CI, confidence interval; OR, odds ratio

Periodontitis	Adverse outcome, 60 (41.4%)	No adverse outcome, 85 (58.6%)	*P*-value, OR (CI)
Present	30 (56.6%)	23 (43.3%)	<0.005^†^, 2.69 (1.343-5.409)
Absent	30 (32.6%)	62 (67.4%)	

## Discussion

The prevalence and impact of periodontal disease among pregnant women vary across regions but consistently highlight its significance as a public health concern. In this study, the prevalence of periodontitis increased with age, cesarean delivery, hypertension, and diabetes, aligning with findings reported in Ethiopia [12], where a 38.8% prevalence was observed and illiteracy, low socioeconomic status, and lack of antenatal care were emphasized as key risk factors. Similarly, a 24% prevalence was observed in Sudan [11], with no significant association with age or parity, highlighting regional differences in influencing factors. Further understanding was extended by demonstrating the direct impact of maternal periodontal disease on neonatal outcomes, linking poor maternal oral health to low-birth-weight infants in Nepal [8]. Association between oral health (periodontal diseases) with adverse pregnancy outcomes (preterm birth or low birth) were reported in a series of studies [4,5,8,18,20-22], which is similar to this study where pregnancy periodontitis was statistically associated with adverse birth outcomes. In a systemic review and meta-analysis of randomized clinical trials, the primary outcome revealed a significant association between periodontitis and adverse pregnancy outcomes. The secondary outcome further highlighted the impact of periodontal disease on complications such as low birth weight, preterm birth, and intrauterine growth restriction, showing that treatment for periodontal disease can significantly reduce these risks [23]. While the precise mechanisms underlying this association are still under investigation, it is hypothesized that oral lesions may induce bacteremia, leading to pathogen transfer to the gravid uterus and cervical-vaginal tissues. This can trigger a systemic inflammatory response involving prostaglandins and cytokines, which, in turn, activate immune cells in the uterus, potentially contributing to adverse pregnancy outcomes [10,24-27]. Some studies have demonstrated that, regardless of the exact mechanism, periodontal treatment specifically scaling and root planning in pregnant individuals has led to improved pregnancy outcomes [2,18,28]. A cross-sectional study conducted in Northern Tanzania found a significant association between periodontal diseases and the risk of adverse pregnancy outcomes. Specifically, the study reported that periodontal diseases are linked to increased odds of low birth weight (adjusted odds ratio [AOR] = 2.41; 95% CI: 1.34-4.33) and preterm birth (AOR = 2.32; 95% CI: 1.33-4.27) [29]. In Nepal, very few studies have been conducted compared globally. A study done in the Terai region of Nepal found a statistically significant association between periodontitis and low birth weight (*P *< 0.0001), which is consistent with the findings of our study [8]. Another prospective cohort community-based study conducted in rural Nepal also identified gingival inflammation in pregnant women as an independent risk factor for preterm birth [9]. This reinforces the significance of periodontal health in influencing adverse pregnancy outcomes, aligning with the results of the current study. However, no associations were observed between any other explanatory variables even for pregnancy-induced morbid conditions like diabetes and adverse pregnancy outcomes contrasting with the findings of the reviews [2,25]. The study had several limitations. A full-mouth examination was omitted due to time constraints and potential inconvenience to participants. The presence of calculus might have led to inaccuracies in measuring pocket depth and attachment loss, and the exclusion of certain groups (e.g., women with alcohol or tobacco use, and specific health conditions) limited the generalizability of the findings. The small sample size of 145 participants may not have had enough statistical power to detect subtle associations with other explanatory variables. Additionally, the cross-sectional design only shows associations and does not establish causality. Uncontrolled confounding variables, such as diet socioeconomic status, or oral hygiene practices could have influenced the results. There was also potential measurement bias in diagnosing periodontitis, as it may have been subject to some subjectivity. However, the significant association with periodontitis suggests it may be a more crucial factor influencing pregnancy outcomes than other variables.

## Conclusions

This study highlights the significant association between maternal periodontitis and adverse pregnancy outcomes, such as low birth weight and preterm birth, consistent with global and regional findings. Despite limitations, including a small sample size, restrictive exclusion criteria, and potential measurement biases, the results emphasize the importance of maternal oral health. Improving periodontal care during pregnancy could reduce risks to both mothers and infants. It is recommended that prenatal care incorporate regular periodontal screenings and treatments. Future research with larger, diverse samples and a longitudinal design is needed to further explore the causal relationship between periodontitis and pregnancy complications.

## Figures and Tables

**Figure 1:**
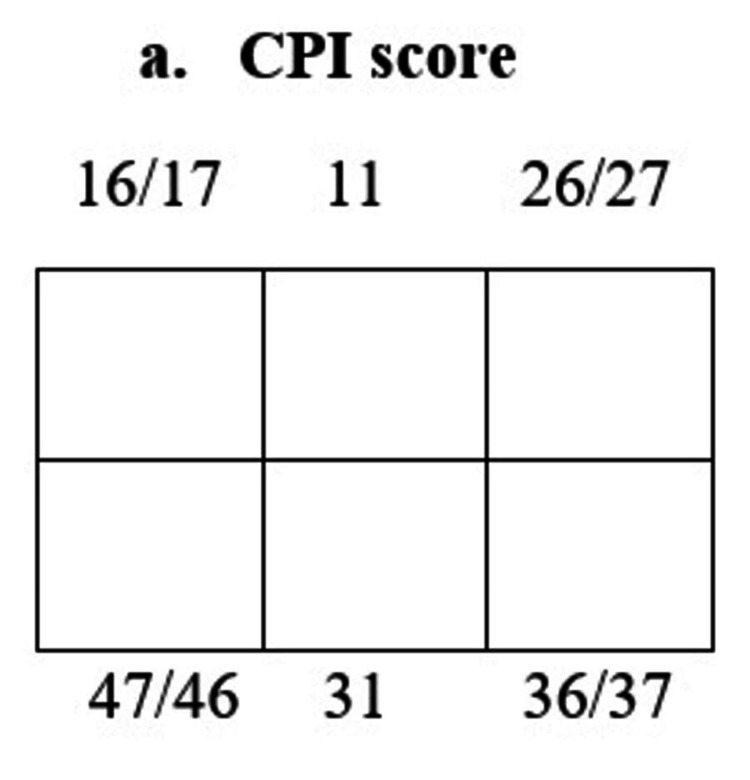
CPI score

**Figure 2:**
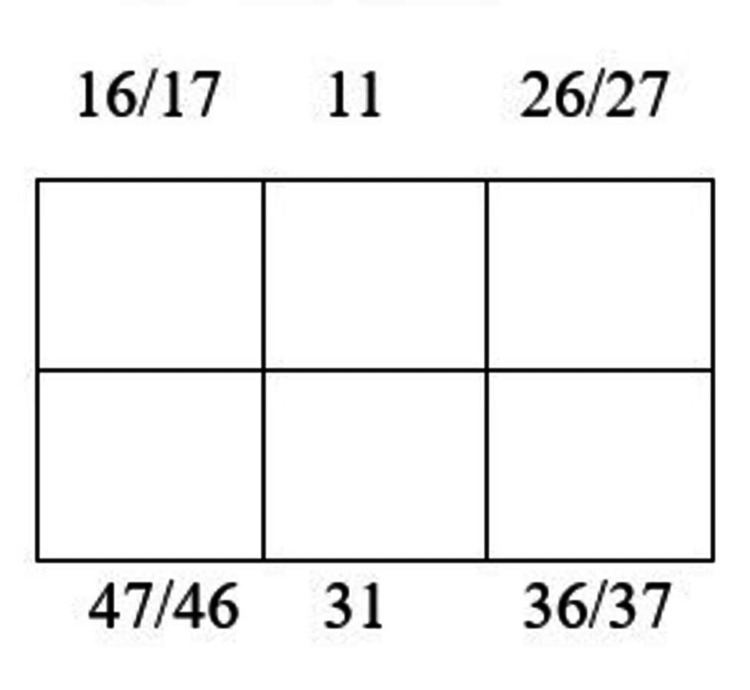
LOA Table

## Appendices

Appendix: Questionnaire

I.D.:

A. Socio-Demographic Information

1.      Age: …………in completed years

2.      Educational status

                                i.            Illiterate   

                              ii.            Literate, If literate,

□ Can read and write, □ Primary level, □ Secondary school, □ Higher secondary school and   □ Bachelor and above

B. Obstetric history-related information

3. No of previous deliveries of respondent □ One, □Two, □ More…………. □No

C.  Morbid status

4. Hypertension: □Yes □ No

                     If yes, □pregnancy-induced   □non-pregnancy induced

5. Diabetes:   □ Yes □No

                  If yes, □ pregnancy-induced □ non-pregnancy induced

6. Presence of Urinary Tract Infection:          □ Yes □ No

7. Thyroid disorder:    □ Yes □No

 If yes, □ pregnancy-induced   □ non-pregnancy induced

8. Other chronic disorders:  □ Yes □ No

                   If yes, specify…………

D. Information Related to Periodontitis

Periodontitis (CPI)

0                      Healthy

1                      Bleeding observed directly or by using a mouth mirror /after probing

2                      Calculus detected during probing but all the black bands on the probe were visible

3                      Pocket 4-5mm (gingival margin within the black band on the probe)

4                      pocket 6mm or more 9 black bands on the probe not visible)

a.      CPI score

  b.      Loss of Attachment

0                    Loss of attachment 0-3mm (CEJ not visible and CPI score 0-3)

If CEJ is visible and CPI score is 4 or if the CEJ is visible then scoring

1                    Loss of attachment 4/5mm (CEJ within the black band)

2                    Loss of attachment 6/8mm (CEJ between the upper limit of the black band and the 8.5mm ring)

3                    Loss of attachment 9-11mm (CEJ between the 8.5mm and 11.5mm rings)

4                    Loss of attachment 12mm or more (CEJ beyond the 11.5mm ring)

E. Adverse Pregnancy Outcome:

9. Mode of delivery:   □ Normal vaginal delivery        □ Cesarian section

10. Gestational age per week of Newborn: ………………….

11. Low Birth Weight ……………gms

12. Small for gestational age = less than the 10th percentile of gestational age

                                             □Yes □ No

13. Intrauterine growth retardation      □ Yes □ No

14. Preterm birth:   □ Yes □ No

15. Congenital anomaly: □ Yes □ No

                                       If yes specify…………….

16. Fetal death (miscarries/stillbirth) □ Yes □ No
